# Molecular, genetic and epigenetic pathways of peroxynitrite-induced cellular toxicity

**DOI:** 10.2478/v10102-009-0020-4

**Published:** 2009-12-28

**Authors:** Ahmet Korkmaz, Sukru Oter, Melik Seyrek, Turgut Topal

**Affiliations:** 1 Department of Physiology, Military Medical Faculty, Gulhane Military Medical Academy, Ankara, Turkey; 2 Department of Pharmacology, Military Medical Faculty, Gulhane Military Medical Academy, Ankara, Turkey

**Keywords:** antioxidants, cytokines, epigenetic, nitrosative stress, oxidative stress, peroxynitrite

## Abstract

Oxidative stress plays a key role in the pathogenesis of cancer and many metabolic diseases; therefore, an effective antioxidant therapy would be of great importance in these circumstances. Nevertheless, convincing randomized clinical trials revealed that antioxidant supplementations were not associated with significant reduction in incidence of cancer, chronic diseases and all-cause mortality. As oxidation of essential molecules continues, it turns to nitro-oxidative stress because of the involvement of nitric oxide in pathogenesis processes. Peroxynitrite damages via several distinctive mechanisms; first, it has direct toxic effects on all biomolecules and causes lipid peroxidation, protein oxidation and DNA damage. The second mechanism involves the induction of several transcription factors leading to cytokine-induced chronic inflammation. Finally, it causes epigenetic perturbations that exaggerate nuclear factor kappa-B mediated inflammatory gene expression. Lessons-learned from the treatment of several chronic disorders including pulmonary diseases suggest that, chronic inflammation and glucocorticoid resistance are regulated by prolonged peroxynitrite production.

**LIST OF ABBREVIATIONS**
AP-1: Activator Protein-1
CAT: Catalase
COPD: Chronic Obstructive Pulmonary Disease
COX-2: Cyclooxygenase-2
Cu/Zn-SOD: Cupper/Zinc Superoxide Dismutase
DNMT: DNA Methyltransferase
EC-SOD: Extracellular Superoxide Dismutase
eNOS: Endothelial Nitric Oxide Synthase
IL-1β: Interleukin-1beta
IL-8: Interleukin-8
iNOS: Inducible Nitric Oxide Synthase
GR: Glucocorticoid Receptor
GSH-Px: Glutathione Peroxidase
HDAC: Histone deacetylase
HAT: Histone Acetyl Transferase
HOPE: The Heart Outcome Prevention Evaluation Trial
MMP: Matrix Metalloproteinase
Mn-SOD: Manganese Superoxide Dismutase
NAC: N-Acetlycysteine
NAD^+^: Nicotinamide Adenine Dinucleotide
NF-κB: Nuclear Factor-kappa B
NO: Nitric Oxide
NO_2_·: Nitrogen Dioxide Radical
NOS: Nitric Oxide Synthase
O_2_·−: Superoxide Anion Radical
OH·: Hydroxyl Radical
ONOO^−^: Peroxynitrite
PARP: Poly(ADP Ribose) Polymerase
PG: Prostaglandin
PMA: Phorbol 12-Myristate 13-Acetate
ROS: Reactive Oxygen Species
SOD: Superoxide Dismutase
TNF-ɑ: Tumor Necrosis Factor-alpha

## Introduction

The free radical theory of aging has gained acceptance and has matured in the past 50 years since it was proposed by Harman in 1956 (Harman, 2003; 2006). Since not only free radicals lead to damage of biomolecules but also other cellular reactive by-products of normal metabolism, the theory has been extended to the ‘oxidative damage theory’ which suggests that reactive oxygen species (ROS) are responsible for the accumulation of age-related cellular debris, and that this damage is an important contributor not only for aging but also for chronic diseases (de Grey, 2006).

In theory, antioxidant interventions should modify the biochemical and molecular events causing cancer and chronic diseases, correct physiological changes responsible for symptoms and signs of several cancers, and decrease the susceptibility to chronic diseases and cancer. Therefore, in excess of 70% of the American population uses dietary supplements daily, of which antioxidant vitamin and mineral supplements are the most common (Woo, 2007). The idea that the pathophysiologic processes caused exclusively by oxygen-derived free radicals could presumably be alleviated by conventional antioxidants such as vitamin E or C and/or intracellular enzymatic antioxidants seems worthy. Nevertheless, several studies found that the antioxidants had little effect in terms of preventing several cancers and chronic diseases.

In the Physicians' Health Study, data from 83,639 US male physicians, of whom 29% were taking either vitamin E, vitamin C, or multivitamin supplements on a self-selected basis, was evaluated (Muntwyler *et al*., 2002). The authors concluded that these supplements were not associated with a significant reduction in total cardiovascular diseases or coronary heart disease mortality. Another prospective study reported that vitamins E and C and certain carotenoids did not reduce the risk of stroke in 43,738 men 40 to 75 years old with no cardiovascular disease or diabetes (Ascherio *et al*., 1999). The Heart Outcome Prevention Evaluation (HOPE) trial (Lonn *et al*., 2002), a randomized controlled trial in patients 55 years or older who had cardiovascular disease or diabetes, revealed that taking 400 IU vitamin E daily for an average of 4.5 years was without influence on cardiovascular outcomes or nephropathy. A randomized, double-blind, placebo-controlled trial with vitamin E interestingly resulted in increased blood pressure in type 2 diabetic patients (Ward *et al*., 2007). The authors concluded that the mechanism for this increase remains unknown; however, it appears to be independent of changes in oxidative stress. Additionally, a meta-analysis of a large body of published data revealed that antioxidant vitamins do not significantly reduce risk of cardiovascular death (Vivekananthan *et al*., 2003).

The lung is the only organ in the entire human architecture, which has the highest exposure to atmospheric oxygen. Owing to its large surface area and blood supply, the lung is susceptible to oxidative injury by virtue of myriads of reactive forms of oxygen species and free radicals. Exogenous oxidants and increased oxygen burden in lung due to accumulation of inflammatory cells in the lower respiratory tract, including macrophages and neutrophils are generally accepted as initial step in patients with acute respiratory distress syndrome, idiopathic pulmonary fibrosis and chronic obstructive pulmonary disease (COPD)(Rahman and MacNee, 1996; Wallaert *et al*., 1990). Free radical reactions have been suggested to play a contributory role in the fibrogenesis either directly or through inflammatory responses (Poli and Parola, 1997). Tissue injury almost invariably leads to oxidative stress. In theory, antioxidant interventions should modify the biochemical and molecular events causing COPD correct physiological changes responsible for symptoms and signs of COPD and reduce the susceptibility to complications associated with COPD (Kirkham and Rahman, 2006).

Vitamin A, C and E, β-carotene, N-acetylcysteine (NAC) and its derivative erdosteine are the most frequently used antioxidants. Among them NAC, which was introduced as a mucolytic agent for COPD more than 50 years ago, is a well-known antioxidant and has been extensively studied in both experimental and clinical studies. In experimental studies, NAC and erdosteine, generally reduce lung injury in many animal models of diseases (Aitio, 2006; Moretti and Marchioni, 2007).

Several randomized-controlled studies have shown that 600 mg NAC given daily reduce exacerbations of COPD (Boman *et al*., 1983; Pela *et al*., 1999; Rasmussen and Glennow, 1988). Most investigations of the role of antioxidant substances in COPD, however, have been inconclusive because they were short-term and only assessed immediate effects (Riccioni *et al*., 2007). Moreover, in acute exacerbations of COPD, addition of NAC to standard treatment did not modify the outcome (Black *et al*., 2004). A recent extensive multi-centre study (BRONCUS), where the patients were followed for 3 years, reported that at least the dose of NAC 600 mg daily is ineffective at prevention of deterioration in lung function and prevention of exacerbations in patients with COPD (Decramer *et al*., 2005).

Bjelakovic *et al*. (Bjelakovic *et al*., 2004) examined a series of studies (170,525 participants) and found that neither antioxidant vitamins singly (e.g., vitamin E and β carotene) nor several combinations (e.g., β carotene, vitamin C and E) showed effect on the incidence of gastrointestinal cancer. Lawson *et al*. (Lawson *et al*., 2007) conducted a prospective observational study and investigated the association between multivitamin use and prostate cancer risk in 295,344 men enrolled in the National Institutes of Health (NIH) – AARP Diet and Health Study. The authors found that use of multivitamins more than seven times per week, when compared with never use, was associated with a doubling in the risk of fatal prostate cancer. A recent prospective cohort of 77,721 men and women aged 50–76 years declared that supplemental multivitamins, vitamin C and E were not associated with a decreased risk of lung cancer. Moreover, supplemental vitamin E was associated with a small increased risk (Slatore *et al*., 2008).

The results are in accord with the results of systematic reviews and meta-analyses of randomized clinical trials examining the efficacy of antioxidant supplements on all-cause mortality. A meta-analysis found that in 11 trials, high doses of vitamin E resulted in slightly but significantly higher rate of all-cause mortality when compared with placebo (Miller *et al*., 2005). Another systematic review and meta-analysis that included 68 randomized trials with 232,606 participants (385 publications) also showed that antioxidant vitamins may increase all-cause mortality (Bjelakovic *et al*., 2007).

## Why antioxidant vitamins do not prevent chronic diseases?

A possible explanation for the potential negative effect of antioxidant supplements might be that ROS in moderate concentrations are essential mediators of reactions by which the body gets rid of unwanted cells. Thus, if administration of antioxidant supplements decreases free radicals, it may interfere with essential defensive mechanisms for ridding the organism of damaged cells, including those that are injured, precancerous and cancerous (Linnane *et al*., 2007). Moreover, antioxidants are potential electron donors and they exhibit both reduced and oxidized forms. Once they donate an electron to neutralize a free radical, they are transformed from a reduced to an oxidized state. Usually, the oxidized form will be regenerated to the reduced state through the mechanism known as redox reaction or recycling. Since these antioxidants are electron donors and exhibit redox reactions, their oxidized forms also can oxidize other molecules, thus, classical antioxidants are often reported to be pro-oxidants (Beckman and Koppenol, 1996; Bjelakovic *et al*., 2007; Pacher *et al*., 2007).

Another possible explanation might be that the studies were conducted in middle- and high income countries among populations already well saturated with vitamins. The American diet provides 120% of the recommended dietary allowances for antioxidant vitamins and dietary vitamin E deficiency has never been reported in the United States (Herbert, 1994). Our diets typically contain safe levels of vitamins, but high-level antioxidant supplements could potentially upset an important physiologic balance (Salganik, 2001). Nevertheless, there is no doubt that oxidative stress is often an early and key event that activates numerous pathways involved in several cancer and development of chronic diseases including type II diabetes, hypertension, atherosclerosis and inflammatory lung diseases. On the basis of published literature, a novel molecular approach to resolve the apparent controversy and to protect cells from oxidative stress is warranted (Ceriello, 2006).

### Nitro-oxidative stress: an explanation for the controversy

A theoretical explanation for the failure of antioxidant vitamins to protect against disease relates to the fact that with the discovery of nitric oxide (NO) and nitric oxide synthase (NOS) family, oxidative stress became more complex then previously realized. A vital, ubiquitous molecule, NO, has numerous roles in regulating physiological processes. Because of affinity of NO for the superoxide anion radical (O_2_·^−^), neither enzymatic nor pharmacologic levels of conventional antioxidants successively compete with NO for O_2_ ·^−^; as a result, high peroxynitrite (ONOO^−^) levels follow (Beckman and Koppenol, 1996). Therefore, the oxidative stress theory has expanded into new areas, particularly after the involvement of ONOO^−^ as a causative reactant (Pacher *et al*., 2007).

**Figure 1:**
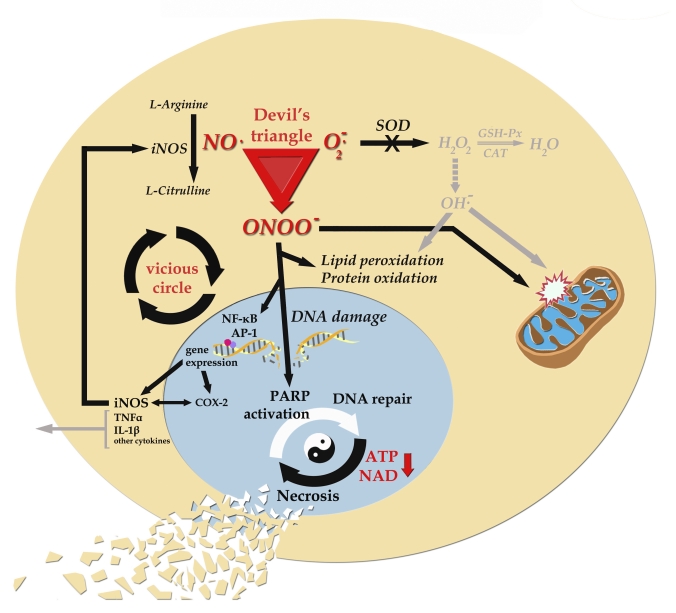
Basic mechanism of peroxynitrite-induced toxicity and related pathways (Korkmaz, 2008b). Oxidative stress is often an early and key event that activates numerous pathways involved in several cancer and development of chronic diseases. If the causative agent (e.g., hyperglycemia, cigarette smoking, UV lights, and chemical toxicants) persists, eventually iNOS is activated and ONOO^−^ is formed. By then, cellular stress is transformed from oxidative only to nitro-oxidative. ONOO^−^ exerts its harmful effects directly and indirectly. It causes activation of transcriptional factors leading to pro-inflammatory gene expression. During this process, nitro-oxidative stress also involves an inflammatory response. Interactions between transcriptional factors and pro-inflammatory products lead to a vicious cycle of damage. The cytokines spread the inflammatory signals through the circulation. Unless excess O2·^−^ and iNOS-derived NO production are terminated, this mechanism continues to propagate damage within cell. Moreover ONOO^−^ directly damage all macromolecules including lipids, proteins and DNA. ONOO^−^-induced DNA damage is sensed by DNA repair enzymes, in particular poly(ADP ribose) polymerase (PARP). In presence of severe genomic damage, overactivation of PARP causes cellular NAD^+^ and ATP depletion by attempting a repair process. This drives cells into an energy crisis eventually leading to necrosis. This futile mechanism, so-called “suicide hypothesis” of PARP activation, is reportedly involved in many diseases related to nitro-oxidative stress. Since the mitochondrion has its own DNA and PARP enzyme, this pathophysiologic process also takes place within the mitochondrion. It is well known that, both oxygen and nitrogen-based radicals are prone to directly damage this organelle. Consumption of the majority of NAD^+^ by PARP also slows the rate of glycolysis and mitochondrial respiration, and eventually leads to cellular dysfunction and death.

It is presumed that initially ROS production reduced the endothelial NOS (eNOS)-derived NO within endothelial cells while activating inducible NOS (iNOS) which causes almost a 1,000-fold higher NO production than eNOS does under physiologic circumstances. iNOS is predominantly expressed in inflammatory cells such as macrophages, although epithelial cells from affected tissues also express the enzyme. Intensified expression of iNOS has been detected in virtually all cell types tested including macrophages, fibroblasts, chondrocytes, osteoclasts, and epithelial cells and results in the production of large amounts of NO in animals and patients with inflammatory diseases (Cooke and Davidge, 2002; Moncada *et al*., 1991; Stockklauser-Farber *et al*., 2000; Weidig *et al*., 2004). The level of iNOS expression is well correlated with the degree of inflammation. A controversy arises from observations reporting both cytotoxic and cytoprotective effects of NO. In cases where NO was found cytotoxic, it was questioned whether NO, directly or indirectly, or through the formation of more reactive species such as the ONOO^−^ exerted these effects. Thus, it is believed that oxygen-based free radicals are only an initial step in the pathophysiologic mechanisms of aging and chronic diseases (Dedon and Tannenbaum, 2004). The combination of elevated NO plus excess O_2_ ·^−^ with the formation of high levels of ONOO^−^ is the proverbial intracellular “devil's triangle” (Figure 1).

### The “devil's triangle” changes the nature of oxidative stress

In the last two decades, NO was found, when produced in excess, to be a toxic molecule which damages and even kills cells. In the presence of excess O_2_ ·^−^ and abundant NO, this latter vital molecule shows its dark side. Neither O_2_ ·^−^ nor NO is particularly toxic by themselves because there are efficient means to minimize their accumulation (Beckman and Koppenol, 1996). Thus, O_2_ ·^−^ is rapidly removed by the superoxide dismutases (SOD) with the isoenzymes of this molecule being located in the mitochondria (Mn-SOD), cytosol (Cu/Zn-SOD), and extracellular (EC-SOD) compartments. Physiologically, NO is removed by its rapid diffusion through tissues into red blood cells (Joshi *et al*., 2002), where it is quickly converted to nitrate by a reaction with oxyhemoglobin. This limits the biological half-life of NO *in vivo* to less than a second. However, when both O_2_ ·^−^ and NO are generated within a few molecular diameters of each other, they combine spontaneously to form ONOO^−^ in a diffusion-limited reaction. Basically, every time NO and O_2_ ·^−^ collide, they form ONOO^−^. NO is the only known biological molecule that reacts faster with O_2_ ·^−^ and is produced in such high concentrations that it out-competes endogenous SOD; hence, the creation of the “devil's triangle” (Figure 1). Consequently, from a biological viewpoint, the reaction of O_2_ ·^−^ with NO to form ONOO^−^ is inevitable. Any of several antioxidants may have beneficial effects in these acute situations (e.g., acute lung injury, ischemia-reperfusion, myocardial infarction, exogenous inflammatory agent-induced acute inflammation). However, in case of chronic oxidative stress, once iNOS is totally activated, ordinary antioxidants provide little protection due to massive ONOO^−^ generation. Thus, in case of chronic oxidative stress such as chronic hyperglycemia, dyslipidemia, tobacco smoking, exposure to allergens, prolonged drug use which are known to produce oxidative damage, conventional antioxidants become ineffective (Korkmaz *et al*., 2008b).

## Consequences of peroxynitrite production

Once ONOO^−^ forms, it can act via several distinct mechanisms; first it has direct toxic effects leading to lipid peroxidation, protein oxidation and DNA damage. The second mechanism involves the induction of several transcription factors including nuclear factor-kappa B (NF-κB) and activator protein-1 (AP-1) leading to cytokine-induced chronic inflammation (Figure 1). This cascade, once activated, causes the release of pro-inflammatory cytokines including tumor necrosis factor-α (TNF-α) and interleukin-1β (IL-1β), and a variety of chemokines which induces widespread inflammation. During this process, several adhesion molecules and monocyte chemoattractant proteins also become involve widening the inflammatory response and vascular events (Forstermann and Munzel, 2006; Pacher *et al*., 2007).

A direct toxic effect of ONOO^−^ at the site of its production involves an intriguing process which decides the fate of cells. ONOO^−^ is per se not a radical but is a powerful nitrosating agent. ONOO^−^ interacts with and covalently modifies all major types of biomolecules including membrane lipids, thiols, proteins and DNA (Demicheli *et al*., 2007; Pacher *et al*., 2007). In addition, ONOO^−^ can yield the hydroxyl (OH·) and nitrogen dioxide (NO_2_ ·) radicals (less than 30% yield). Although this is a minor process in biology, OH· is a potent reactant and oxidizes relevant targets including amino acids (tyrosine, phenylalanine, histidine), carbohydrates and lipids (Alvarez and Radi, 2001; 2003). The generation of ONOO^−^ also decreases the availability of NO for G-protein stimulation and vasodilatation, thus further contributing to endothelial dysfunction leading to elevated blood pressure. In addition, ONOO^−^ can inhibit SOD as well as other antioxidant molecules and systems, which leads to positive feedback cycles of intracellular oxidant generation and increased radical damage (Szabo, 2003). ONOO^−^ activates matrix metalloproteinases (MMPs) (Okamoto *et al*., 2001; Wu *et al*., 2001) and triggers the expression of selectins and cellular adhesion molecules via enhancement of NF-κB activation thereby promoting pro-inflammatory responses.

The mutagenic properties of ONOO^−^-induced modified products have also been determined (Juedes and Wogan, 1996; Whang *et al*., 1998). Several studies have shown that NO itself does not induce DNA single-strand breaks in vitro in plasmid DNA (Masuda *et al*., 2002; Tamir *et al*., 1996), whereas exposure of plasmid DNA to pre-formed ONOO^−^ (Yoshie and Ohshima, 1997) or NO plus O_2_ ·^−^ generated concurrently induces such strand breaks (Chaturvedi *et al*., 1998). Single strand breakage can be induced by treatment with very low concentrations of ONOO^−^ indicating that this agent is a potent inducer of this type of damage to DNA (Yermilov *et al*., 1996). DNA cleavage caused by ONOO^−^ was observed at almost every nucleotide, with a small preference for guanine residues. Furthermore, it has been reported that ONOO^−^ inactivates several enzymes that are critically involved in the repair of DNA damage. These observations suggest additional pathways by which ONOO^−^ may be associated with not only elevated DNA damage but also impairment of DNA repair capacity (Chien *et al*., 2004).

### A crucial step: poly(ADP ribose) polymerase activation

ONOO^−^ induces both apoptosis and necrosis of cells. More highly elevated exposure of this agent is associated with necrosis as opposed to apoptosis (Levrand *et al*., 2006; Szabo, 2003; Virag *et al*., 2003). In this mechanism, activation of the DNA repair enzyme poly(ADP ribose)polymerase-1 (PARP-1), a member of PARP enzyme family, mediates ONOO^−^-induced necrosis. PARP-1 detects and signals DNA strand breaks induced by a variety of genotoxic insults including ionizing radiation, alkylating agents, oxidants (essentially OH·, ONOO^−^), and free radicals (Korkmaz *et al*., 2005; Korkmaz *et al*., 2007; Korkmaz *et al*., 2006; Szabo, 2006; Yaren *et al*., 2007). Upon binding to DNA, strand breaks occur and PARP transfers ADP-ribose units from the respiratory coenzyme nicotinamide adenine dinucleotide (NAD^+^) to various nuclear proteins. From a physiological view point, PARP-1 activity and poly(ADP-ribosyl)ation reactions are implicated in DNA repair processes, the maintenance of genomic stability, the regulation of gene transcription, and DNA replication. An important function of PARP-1 is to allow DNA repair and cell recovery under conditions associated with a low level of DNA damage. In case of severe DNA injury, overactivation of PARP-1 depletes the cellular stores of NAD^+^, an essential cofactor in principal energy production mechanisms including the glycolytic pathway, the tricarboxylic acid cycle, and the mitochondrial electron transport chain. As a result, the loss of NAD^+^ leads to a marked reduction in the cellular pools of ATP, resulting in cellular dysfunction and death via the necrotic pathway (Szabo, 2003; Virag *et al*., 2003). This is known as the “suicide hypothesis” of PARP activation and seems to be a regulatory mechanism to eliminate cells after irreversible DNA injury. A vast amount of experimental evidence has established that the PARP-1 pathway of cell death plays a pivotal role in tissue injury and organ dysfunction in numerous disease processes (de la Lastra *et al*., 2007; Korkmaz *et al*., 2006; 2008a).

### A novel mechanism of peroxynitrite-induced toxicity; lessons-learned from treatment of patients with chronic obstructive pulmonary disease (COPD)

One of the major problems in the treatment of COPD is glucocorticoid resistance. Although inhaled glucocorticoids are highly effective in asthma, they provide relatively little therapeutic benefit in COPD, despite the fact that active airway and lung inflammation is present. This may reflect that the inflammation in COPD is not suppressed by glucocorticoids, with no reduction in inflammatory cells, cytokines or proteases in induced sputum even with high doses of inhaled and oral glucocorticoids (Loppow *et al*., 2001). Furthermore, histological analysis of peripheral airways of patients with severe COPD shows an intense inflammatory response, despite treatment with high doses of inhaled glucocorticoids (Hogg *et al*., 2004). There is increasing evidence for an active steroid resistance mechanism in COPD, as glucocorticoids fail to inhibit cytokines (e.g., IL-8 and TNF-α) that they normally suppress (Culpitt *et al*., 1999).

The predominant effect of glucocorticoids is to switch off multiple inflammatory genes (encoding cytokines, chemokines, adhesion molecules and inflammatory enzymes) that have been activated during the chronic inflammatory process. The increased expression of most of these inflammatory proteins is regulated at the level of gene transcription through the activation of pro-inflammatory transcription factors, such as nuclear NF-κB and AP-1. The molecular pathways involved in regulating inflammatory gene expression are now being delineated and it is now clear that chromatin remodeling and a variety of epigenetic mechanisms play a critical role in the transcriptional control of genes. Stimuli that switch on inflammatory genes do so by changing the chromatin structure of the inflammatory gene, whereas glucocorticoids reverse this process.

Recent evidence suggests that ONOO^−^-induced histone modifications may play a role in the mechanism of glucocorticoid resistance (Barnes *et al*., 2004; Ito *et al*., 2004; Mroz *et al*., 2007). Therefore, ONOO^−^ complicates the pathophysiology and treatment of COPD via several manners; it causes severe nitro-oxidative stress and harms all biomolecules including lipids, proteins, DNA and activates PARP-1 (Hageman *et al*., 2003). It exaggerates inflammation via enhancing NF-B pathway and it directly causes glucocorticoid resistance by blocking glucocorticoid-dependent inflammatory gene silencing (Barnes *et al*., 2004; Mroz *et al*., 2007).

## A summary of epigenetic regulation of gene expression

The term epigenetic describes the study of inheritable alterations in gene expression that occur in the absence of changes in genome sequence. This is in contrast to genetics, which deals with the transmission of information based on differences in DNA sequence. Therefore, epigenetic gene regulation requires molecular mechanisms that encode information in addition to the DNA base sequence and can be propagated through mitosis and meiosis. Our current understanding of epigenetic regulation of gene expression involves basically two classes of molecular mechanisms: histone modifications and DNA methylation. A variety of enzymes are involved in this process including most importantly histone deacetylases (HDACs), histone acetyl transferases (HATs) and DNA methyltransferases (DNMTs) (Miremadi *et al*., 2007). Alteration of the structure of chromatin is critical to the regulation of gene expression. Chromatin is made up of nucleosomes, which are particles consisting of DNA associated with an octamer of two molecules each of the core histone proteins (H2A, H2B, H3 and H4), around which 146 base pairs of DNA are wound. In resting conditions, DNA is wound tightly around these basic core histones, excluding the binding of the enzyme RNA polymerase II, which activates the formation of messenger RNA. This conformation of the chromatin structure is described as closed, and is associated with the suppression of gene expression.

DNA methylation is another regulation, in which a cytosine base is modified by a DNMT at the C5 position of cytosine, a reaction that is carried out by various members of a single family of enzymes. CpG islands are CG-rich sequences located near coding sequences and serve as promoters for the associated genes and methylation of CpG sites is maintained by DNMTs. DNA methylation is commonly associated with gene silencing and contributes to transcriptional regulation of tissue-specific genes during cellular differentiation. The methylation status of CpG islands within promoter sequences works as an essential regulatory element by modifying the binding affinity of transcription factors to DNA binding sites. Gene transcription only occurs when the chromatin structure is opened up, with unwinding and properly methylated of DNA so that RNA polymerase II and basal transcription complexes can now bind to the naked DNA to initiate transcription.

The epigenotype can be transmitted from a parent cell to a daughter cell maintaining a specific epigenotype within cell lineages. Thus, the phenotype is a result of the genotype, the specific DNA sequence, and the epigenotype. The genotype must exist in a particular chromatin configuration, the epigenotype, which allows a secondary level of fine control over gene expression. The epigenotype shows far greater plasticity than the genotype, and it has been speculated that epigenetic errors could be a major contributor to human diseases (Jiang *et al*., 2004). Epigenotype is generally accepted as being less stable than the genetic system, and more sensitive to environmental (McLachlan, 2001), nutritional (Gallou-Kabani and Junien, 2005) and chemical toxicants (Bombail *et al*., 2004; McLachlan *et al*., 2001).

### Peroxynitrite-dependent glucocorticoid resistance in the treatment of chronic obstructive pulmonary diseases

**Figure 2:**
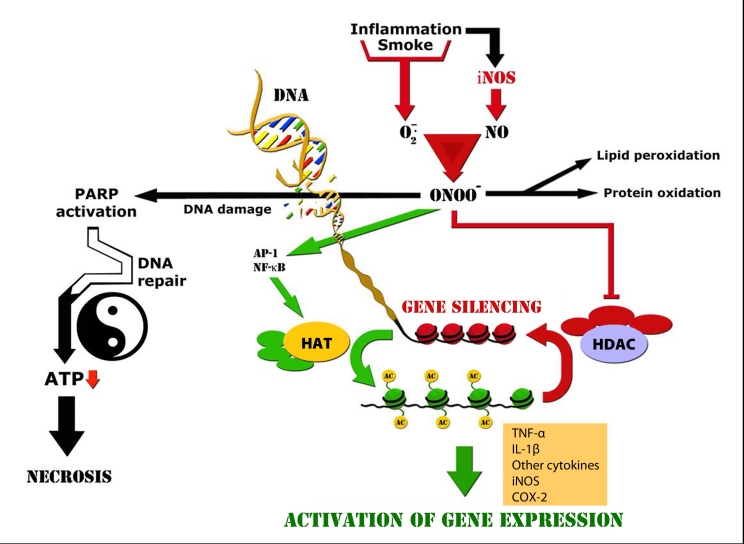
Lessons-learned from treatment of patients with COPD and proposed overall mechanism of ONOO^−^-induced cell toxicity. NF-κB and AP-1 switch on inflammatory genes by inducing several co-activators (e.g., p300/CBP) that have intrinsic HAT activity. Gene transcription only occurs when the chromatin structure is opened up, with unwinding and properly methylated of DNA so that RNA polymerase II and basal transcription complexes can now bind to the naked DNA to initiate transcription. Glucocorticoids switch off multiple inflammatory genes that have been activated by NF-κB and AP-1 during the chronic inflammatory process. Both activation of HDAC and inhibition of HAT may be involved in glucocorticoid-dependent gene silencing. As found in patients with COPD, ONOO^−^ may block the HDAC activity, thereby cause glucocorticoid resistance. This mechanism may partly explain the controversy that antioxidants that only have the capability of scavenging superoxide, but not peroxynitrite may fail in a variety of chronic oxidative stress.

Glucocorticoids produce their effect on responsive cells by activating the glucocorticoid receptor (GR) to directly or indirectly regulate the transcription of target genes. The number of genes per cell directly regulated by glucocorticoids is estimated to be between 10 and 100, but many genes are indirectly regulated through an interaction with other transcription factors and co-activators. Most of the anti-inflammatory actions of glucocorticoids are due to suppression of the actions of AP-1 and NF-κB (Barnes, 2006). The activated GR may directly bind to p300/CBP or other co-activators to inhibit their HAT activity, thus preventing the subsequent histone acetylation and chromatin remodeling and leads to inhibition of AP-1 and NF-κB-induced pro-inflammatory gene expression such as TNF-α, IL-1β and adhesion molecules (Adcock *et al*., 2004). Another mechanism is to reverse this process by deacetylating the hyper-acetylated histones through the recruitment of HDAC-2 to the activated co-activator complex (Ito *et al*., 2006). This process results in rewinding and compaction of DNA, exclusion of RNA polymerase, and suppression of inflammatory gene transcription. This mechanism could account for the anti-inflammatory effect of glucocorticoids in inflammatory diseases (Adcock *et al*., 2004; Figure 2).

Patients with COPD has been shown to have a progressive reduction in total HDAC activity that reflects the severity of the disease (Ito *et al*., 2005; 2006). There is also a reduction in total HDAC activity in peripheral lung, bronchial biopsy specimens, and alveolar macrophages from COPD patients, and this is correlated with disease severity and with increased gene expression of IL-8 (Ito *et al*., 2005). HDAC activity is reduced in alveolar macrophages of cigarette smokers compared to nonsmokers, and this is correlated with increased expression of inflammatory genes in these cells (Ito *et al*., 2001). Importantly, HDAC-2 has been found to mediate the deacetylation of the GR that enables NF-κB suppression (Ito *et al*., 2006). It was suggested that HDAC-2 is a key enzyme involved in the suppression of NF-κB-mediated inflammatory gene expression. Therefore, HDAC-2 reduction is involved in both glucocorticoid resistance and NF-κB-mediated inflammatory gene expression. The importance of this mechanism in glucocorticoid-insensitive COPD disease is emphasized by over-expression of HDAC-2, which restores glucocorticoid sensitivity in primary cells from these patients.

The reasons for the reduction in HDAC, particularly HDAC-2, in COPD are not yet completely understood. However, there is increasing evidence that this may be due to inactivation of the enzyme of nitro-oxidative stress, in particular ONOO^−^ (Marwick *et al*., 2004; Moodie *et al*., 2004; Rahman *et al*., 2004). As mentioned previously, NF-κB is a transcription factor implicated in the transcriptional upregulation of inflammatory genes in response to changes in cellular oxidation-reduction status. Target genes of NF-B in various cell types encode proteins involved in immune, inflammatory and acute-phase responses. Recently, considerable progress has been made in understanding the details of the signaling pathways that regulate NF-B activity, particularly those responding to the pro-inflammatory cytokines TNF-α and IL-1 (Wang *et al*., 2002).

Once in the nucleus, NF-κB undergoes post-translational modifications (e.g., phosphorylation) that increase NF-κB transcriptional activation. These modifications facilitate complexes with co-regulators such as p300/CBP that promote NF-κB transcriptional responses (Perkins *et al*., 1997). For example, phosphorylated p65 is more effective at displacing transcriptionally repressive HDAC complexes such as p50-HDAC from κB enhancers within target genes. p300 has been implicated in this acetylation but other HATs could also be involved (Chen and Greene, 2003). Furthermore, HDACs such as HDAC-3 can deacetylate p65. The balance between HATs and HDACs could influence the transcriptional potential of NF-κB. Other transcription factors are also post-transcriptionally modified. For example p53 can be phosphorylated and acetylated affecting its transcriptional responses (Roy *et al*., 2005). These post-translational modifications could provide a transcriptional “histone code” allowing gene transcription to be targeted to specific genes.

To allow NF-κB as well as the transcriptional machinery access to target genes, chromatin remodeling is required. The acetylation of histone tails surrounding the promoters for NF-κB target genes has been shown. Acetylation of histone tails is regulated by HATs and HDACs similar to NF-B acetylation. Indeed, many NF-κB targeted genes have p300/CBP complexes on their promoters thereby increasing histone acetylation (Chen and Greene, 2003). Moreover, HDAC inhibitors that increase acetylation of proteins require an intact NF-κB signaling pathway to induce cell cycle arrest in human myeloid leukemia cells (Dai *et al*., 2003). Conversely, deacetylation of histones impairs and blocks NF-κB action (Campbell *et al*., 2004).

One confusing problem in NF-κB transcriptional regulation is the contradictory functions of NF-κB in apoptosis and cell survival. Many stimuli give survival responses to cells that are also mediated by NF-κB. For example, an early cellular response to oxidative stress is an activation of antioxidant enzymes, including SOD, glutathione peroxidase (GSH-Px) and catalase (CAT). Likewise, the antioxidant enzyme genes are present in the promoter gene region of κB response elements (Sun and Oberley, 1996). Xu *et al*. described that NF-κB is critical for TNF-α and IL-1β induced Mn-SOD expression (Xu *et al*., 1999). Kiningham *et al*. reported that the induction of Mn-SOD by phorbol 12-myristate 13-acetate (PMA), a well-known inflammatory agent, and cytokines depend on the increased NF-κB activity. They found that simultaneous treatment with PMA, TNF-α and IL-1β synergistically increases Mn-SOD induction through the NF-κB pathway (Kiningham *et al*., 2001). Song *et al*. (2005) reported that oxidative stress increases NF-κB DNA binding activity in an experimental ischemia-reperfusion model. They further revealed that increased expression of SOD and subsequent decrease of oxidative stress promoted the neuroprotective expression profile of NF-κB-related genes resulting in neuroprotection (Song *et al*., 2007). Supportingly, Mattson *et al*. (2000) reported that the NF-κB subunit plays a role in regulating neuronal survival in neurodegenerative diseases, probably by targeting genes such as Mn-SOD.

On the other hand, iNOS and cyclooxygenase-2 (COX-2) are also regulated by NF-κB and lead to excess prostaglandin (PG) synthesis. It is known that NF-κB represents one of the most relevant signaling pathways that mediates the expression of iNOS following many extracellular mediators, including endotoxin and inflammatory cytokines (Mollace *et al*., 2005). How the same transcription factor, NF-κB, can regulate several genes with juxtaposed and/or opposed functions? NF-κB may have diverse effects over gene expression and may have several faces as recently reviewed by Graham and Gibson (Graham and Gibson, 2005). The possible reason of this that subunits of NF-κB and/or different combinations of activated subunits may cause a variety of epigenetic actions upon the histone code of targeted genes and lead diverse variety of gene expression. Recently, Song *et al*. (2007) reported that reduced oxidative stress promotes NF-κB-mediated neuroprotective gene expression and alleviates oxidative stress after transient focal cerebral ischemia.

## Concluding remarks

Since antioxidant supplements can reduce the oxidative stress, they may help in acute oxidative stress. In case of chronic circumstances (e.g., chronic hyperglycemia, tobacco smoking, exposure to chemical and dietary toxins), oxidative stress turns nitro-oxidative stress and antioxidant supplements can no longer reduce oxidative stress and redox status of cells are perturbed. There is no doubt that, nitro-oxidative stress plays a crucial role in virtually all acute and chronic inflammatory circumstances irrespective to causative agents and promotes NF-κB-mediated inflammatory gene expression by both inducing NF-κB itself and reducing HDAC levels of inflammatory genes. Therefore, a possible beneficial drug should exert not only antioxidant efficacy but also iNOS inhibitory and/or ONOO^−^ scavenging properties. This kind of therapeutics may help the patients with chronic inflammatory diseases as an add-on treatment along with steroid or non-steroid anti-inflammatory drugs.
